# Identifying Transfer Learning in the Reshaping of Inductive Biases

**DOI:** 10.1162/opmi_a_00158

**Published:** 2024-09-15

**Authors:** Anna Székely, Balázs Török, Mariann Kiss, Karolina Janacsek, Dezső Németh, Gergő Orbán

**Affiliations:** Department of Computational Sciences, HUN-REN Wigner Research Centre for Physics, Konkoly-Thege Miklós út 29-33., H-1121, Budapest, Hungary; Department of Cognitive Science, Faculty of Natural Sciences, Budapest University of Technology and Economics, Műegyetem rkp. 3., H-1111 Budapest, Hungary; Mozalearn Ltd, 6720, Szeged, Hungary; Centre for Thinking and Learning, Institute for Lifecourse Development, School of Human Sciences, Faculty of Education, Health and Human Sciences, University of Greenwich, Greenwich, SE10 9LS United Kingdom; Institute of Psychology, Faculty of Education and Psychology, Eötvös Loránd University, 1071 Budapest, Damjanich u. 41-43, Hungary; Université Claude Bernard Lyon 1, CNRS, INSERM, Centre de Recherche en Neurosciences de Lyon CRNL U1028 UMR5292, 69500, Bron, France; NAP Research Group, Institute of Psychology, Eötvös Loránd University & Institute of Cognitive Neuroscience and Psychology, HUN-REN Research Centre for Natural Sciences, Budapest, 1071, Hungary; Department of Education and Psychology, Faculty of Social Sciences, University of Atlántico Medio, 35017, Las Palmas de Gran Canaria, Spain

**Keywords:** transfer, metalearning, generalization, non-parametric bayesian modeling, learning to learn, statistical learning, inductive biases

## Abstract

Transfer learning, the reuse of newly acquired knowledge under novel circumstances, is a critical hallmark of human intelligence that has frequently been pitted against the capacities of artificial learning agents. Yet, the computations relevant to transfer learning have been little investigated in humans. The benefit of efficient inductive biases (meta-level constraints that shape learning, often referred as priors in the Bayesian learning approach), has been both theoretically and experimentally established. Efficiency of inductive biases depends on their capacity to generalize earlier experiences. We argue that successful transfer learning upon task acquisition is ensured by updating inductive biases and transfer of knowledge hinges upon capturing the structure of the task in the inductive bias that can be reused in novel tasks. To explore this, we trained participants on a non-trivial visual stimulus sequence task (Alternating Serial Response Times, ASRT); during the Training phase, participants were exposed to one specific sequence for multiple days, then on the Transfer phase, the sequence changed, while the underlying structure of the task remained the same. Our results show that beyond the acquisition of the stimulus sequence, our participants were also able to update their inductive biases. Acquisition of the new sequence was considerably sped up by earlier exposure but this enhancement was specific to individuals showing signatures of abandoning initial inductive biases. Enhancement of learning was reflected in the development of a new internal model. Additionally, our findings highlight the ability of participants to construct an inventory of internal models and alternate between them based on environmental demands. Further, investigation of the behavior during transfer revealed that it is the subjective internal model of individuals that can predict the transfer across tasks. Our results demonstrate that even imperfect learning in a challenging environment helps learning in a new context by reusing the subjective and partial knowledge about environmental regularities.

## INTRODUCTION

One striking aspect of human intelligence is the ability to acquire new knowledge in an extremely data and time-efficient manner, especially if compared to artificial learning agents. The stunning ability of humans and other animals to learn is attributed to the ability to acquire the structural regularities of the environment and utilize this knowledge when facing similar challenges. This phenomenon, called transfer learning (also known as learning to learn or meta-learning), is possible since primate perception doesn’t specialize in processing a stream of unrelated stimuli but exploits the fact that the rules and regularities structuring the environment can effectively generalize to novel situations (Brady et al., 2009; Gershman & Niv, 2010). In other words, learning is an inductive process being constrained by the rules and regularities of the world (Kemp et al., 2007; Tenenbaum et al., 2006), often referred to as structures (Kemp & Tenenbaum, 2008). The structured (and often hierarchical) organization of the environment is reflected in the acquired mental representations. Consequently, learning occurs on multiple levels too: first, on the surface, when the sensory stimuli are processed and learned within the context of a learned structure, then on an abstract (or meta-) level, when hidden structures are picked up (Behrens et al., 2018; Dekker et al., 2022; del Ojo Balaguer, 2018; Harlow, 1949; Kemp & Tenenbaum, 2008; Kóbor et al., 2020; Lake et al., 2017; Mark et al., 2020; Orbán et al., 2008; Raju et al., 2022; Samborska et al., 2022; Tse et al., 2007; Whittington et al., 2018, 2022). Our goal is to understand the computations which enable this dual-level learning.

Discovery of hidden structures is critical since it has the potential to generalize across different domains and can be critical contributors to effective learning (Acuña & Schrater, 2010; Eckstein & Collins, 2021; Schulz et al., 2020). Structured organization of the environment can be identified in specific knowledge domains, such as living species are best described by a tree structure, social structures within a group of primates can be captured by group-like structures, while the recurring characteristics of crop development can be described as cycles, to name a few examples (Kemp & Tenenbaum, 2008). Since these structures are maintained as meta-level or abstract representations, when learning on a particular domain, these structure representations serve as inductive constraints (Griffiths et al., 2010; Kemp et al., 2007; Lucas & Griffiths, 2010; Mark et al., 2020; Tian et al., 2020). Importantly, these constraints both allow learning to be much more data and time-efficient and also bias learning toward existing structure representations (Gershman & Niv, 2015; Lucas et al., 2014; Mark et al., 2020). Such influences of acquired knowledge on current interpretations are formalized in a Bayesian approach of learning as the contribution of the prior. A particularly effective form of prior for generalization over tasks or domains is referred to as hierarchical prior, through which a higher-level prior is identified over an inventory of models, which captures regularities of the structure of the earlier experiences. This high-level prior is in contrast with the low-level prior that establishes expectations for the parameters of a particular model. Critically, while priors are often considered to be given for learning, in transfer learning it is this high-level prior that is learned through experience. We will refer to these high-level structure representations as inductive biases instead of priors to emphasize their role in shaping learning. Existing inductive biases dramatically speed up learning when abstract structures are congruent with a new situation (often referred to as tasks in controlled laboratory settings) to be learned (Behrens et al., 2018; Collins & Frank, 2013; Hennies et al., 2016; King et al., 2019; Kumaran et al., 2016; Lake et al., 2017; Mark et al., 2020; Tse et al., 2007; van Kesteren et al., 2010, 2012, 2013; Wang, 2021; Wu et al., 2022), on the contrary, they can also hinder learning in case of incongruent situations (Collins & Frank, 2013; Farzanfar et al., 2023; King et al., 2019; Lucas et al., 2014; Szegedi-Hallgató et al., 2017; Török et al., 2022; Wolpert & Flanagan, 2016; Wu et al., 2022). Operating on a task with incongruent inductive biases implies the need for updating inefficient inductive biases (Török et al., 2022). These updates are essential components of transfer learning since varying the task might imply the need for updating inductive biases. Despite the essential role of these inductive bias updates within transfer learning, we have limited knowledge on how these inductive biases adapt to novel environmental regularities.

The acquisition of hidden task structures has been most widely investigated in animal research, a topic often referred to as schema research (Harlow, 1949; King et al., 2019; Tse et al., 2007; van Kesteren et al., 2010, 2012, 2013), but humans’ and artificial learning agents’ capability of acquiring environmental regularities has also been investigated (Collins & Frank, 2013; del Ojo Balaguer, 2018; Farzanfar et al., 2023; Gilboa & Marlatte, 2017; Mark et al., 2020; Nelli et al., 2023; Schulz et al., 2020; Whittington et al., 2018; Wu et al., 2022). The key finding of this research is that repeated experiences create a generalisable representation of the underlying structure. These generalisable representations later permit flexible remapping to a new sensory environment which enables accelerated learning on new tasks (Behrens et al., 2018; Farzanfar et al., 2023; Gilboa & Marlatte, 2017; Hennies et al., 2016; Lewis & Durrant, 2011). To acquire these generalisable structure representations, extended exposure (often several weeks) to a specific stimulus set is necessary (Török et al., 2022; Tse et al., 2007), during which general features of the task or the environment are disentangled from the sensory stimuli (Behrens et al., 2018; Farzanfar et al., 2023; Gilboa & Marlatte, 2017; Hennies et al., 2016; Lewis & Durrant, 2011). In summary, previous research which attempted to understand how generalisable representations shape learning mainly focused on the environmental characteristics acquired, maintained and transferred across tasks. However, less emphasis was given to the fact that while inductive biases do reflect environmental regularities, learning is hardly ever perfect, thus transferred inductive biases will be inherently constrained by the fallacies of the learning phase.

Based on this insight, we propose an approach to understand the computations behind transfer learning, which relies on two important characteristics: (i) the acquisition of underlying environmental structures is hardly ever perfect, (ii) learning is always a highly individual process, even within strictly controlled laboratory conditions. Characterizing transfer learning will thus be dependent on being able to characterize the idiosyncratic errors subjects made during their learning efforts. To target this gap, we opt for a tool that is able to describe incomplete subjective task representations and individual inductive biases.

In this study we used a non-trivial sequence learning paradigm to study the inductive biases associated with temporal structure of the data. Temporal inductive biases are ubiquitous in natural phenomena, language, and quite prominently, music, where these inductive biases represent the elaborate, potentially hierarchical temporal structure underlying a scene, a language, or genre. We used a sequential structure that ensured that initial inductive biases differ from the one used in the paradigm (Török et al., 2022), which made it possible to identify the contribution of original inductive biases and permitted the study of newly emerging ones. In order to make the update of inductive biases possible, we trained participants in a specific sequence for eight days (referred to as Training phase) and investigated transfer capabilities in two additional days (Trasfer phase, Alternation test Phase), in which a new sequence is introduced that still retains the high-level structure characteristic of the sequence used during the initial training. With this training schedule, we seek to distinguish two key alternatives of learning. First, when learning occurs only on the level of stimulus sequence, i.e. by acquiring increasingly more complex sequential model as training progresses (Éltető et al., 2022; Török et al., 2022). Second, when learning also occurs at a higher level, i.e. when structure-related knowledge, the inductive bias, is updated too. To avoid the assumption of perfect learning of the task, we track day-by-day gradual development of individual learning (i.e. learning curves) and characterize subjective knowledge about the specific task by inferring individualized task representations that we term an internal model of the task. We use Cognitive Tomography (Houlsby et al., 2013; Török et al., 2022) a machine learning-based tool, to infer individualized internal models from behavioral responses. To capture sequential knowledge in the internal model, we use a nonparametric version of the hidden Markov model, the infinite hidden Markov model (iHMM) (Van Gael et al., 2008). Beam Sampling for the Infinite Hidden Markov Model). First, we identify an initial inductive bias of individuals and track the development of the internal model to assess the evolution of the contribution of the inductive bias to later stages of the experiment. Second, we demonstrate that the dominance of the initial inductive bias is highly reduced upon exposing participants to a new sequence with a structure matching initial training. Next, we show that a second internal model is established in the short time frame of the Transfer phase. We also show that the (Training phase) internal model acquired in the Training phase is not replaced, instead, an inventory of internal models is established. Finally, we demonstrate that the structure of the subjective internal model generalizes from the Training phase to the Transfer phase of the experiment, by identifying the contribution of the updated inductive biases in the internal model acquired under the Transfer phase.

## METHODS

### Experimental Participants

25 healthy, young adults, 22 females, 3 male, aged between 18–22 were trained for the experimental paradigm. The population size ensured sampling the diversity of learning strategies while also identifying consistent strategies in subgroups. Hemisphere dominance was also recorded (i.e. which hand or leg is more dominant during special motor tasks). Nevertheless, since motor responses that were required during the current task relied on the use of two fingers on the right and two fingers on the left hand, we did not include hemisphere dominance or handedness in our analysis.

### Data Collection and Training Schedule

Participants are trained for 10 weeks, one training session took place on each week. Multi-day training design is motivated by two reasons, first, learning and consolidating a non-trivial task structure takes time, second, it allows better characterization of individual differences despite potentially fluctuating individual performances. One session consists of 25 blocks of trials (except the tenth day of training which consists of 20 blocks), one block consists of 85 trials. The first five trials in each block are random, after which the 8 element long alternating sequence of random and deterministic elements (r-d-r-d-r-d-r-d) is repeated 10 times. The deterministic components of the sequence are governed by a specific rule that determines the upcoming observation (out of the four possible) with probability = 1 at each deterministic state. For more details see (Török et al., 2022).

Participants are expected to respond in the shortest possible time by pressing the corresponding button on a QWERTY keyboard according to the appearing stimuli, using their index and middle fingers. RTs for the first five random trials, for incorrect responses, RTs longer than 5000 ms, and RTs longer than the mean +3 standard deviation of the individual were removed from the data before the analysis. Furthermore, RTs shorter than 180 ms were also removed, since according to (Carpenter & Williams, 1995) these come from an alternative distribution. Incorrect responses take 11% of trials overall, while due to the 180 ms lower threshold overall 2.2% of the trials were removed (5.3% on Day 8). Longer than 5000 ms trials take less than 0.001% of the trials, RTs longer than the mean + 3 std account for 1.1% for all the trials.

### Models

The computational models used in this paper were developed and formalized in (Török et al., 2022), here we introduce them only briefly. For a more detailed description see the original publication. We introduced three models: the Cognitive Tomography model, the Ground Truth model, and the Markov model. All three models we used in this paper were probabilistic models, relying on a Bayesian modeling approach of sequential observations. The models consist of two parts: the first part captures each participants’ subjective internal representation regarding the task structure, while the second part links sequential inferences to response times. In particular, the sequential probabilistic models calculate predictive probabilities of the upcoming observation based on earlier observations, and these predictive probabilities are then transformed to response times through the behavioral model, for which we adopted the Linear Ascend to Threshold with Ergodic Rate (LATER) model (Carpenter & Williams, 1995). Models were assessed through their predictive performance, i.e. the correrlation between the measured and predicted reaction times on a held out dataset. The model is focussing on capturing the internal model maintained by individuals. Performance of CT is not interpreted in absolute terms, instead the alternative models are used as reference models that implement specific assumptions about the internal model. Some of the variance not explained by CT can be accounted for by lapses, noise in the recording device, reactive inhibition (Török et al., 2017), or errors in dynamical inference, which are beyond the scope of the current model. The three alternative models differ in both their expressive power and their flexibility.

#### Markov Model.

The Markov model is a first-order Markov model, applying the classical Markov condition, where the prediction for the upcoming observation (*y*_*t*+1_) depends only on the previous observation (*y*_*t*_): *p*(*y*_*t*+1_|*y*_*t*_). Thus, the Markov model is able to capture transitions between adjacent elements of a sequence but not the alternating dynamics of our stimulus sequence. The predictive performance of this model shows the extent to which our participants rely on a first-order dependence structure when doing the task; thus, decreasing Markov performance throughout training reflects decreasing maintenance of the first-order belief.

#### Ground Truth Model.

Through the Ground Truth model we formalise an ideal observer model: *p*(*y*_*t*+1_|*y*_1_, *y*_2_, …, *y*_1_, *y*_*t*_). The eight-state HMM is parametrized such that it corresponds to the true generative model of the sequence of stimuli. As such, the parameters of the Ground Truth model were not inferred from RTs, as opposed to Cognitive Tomography, but were set with 8 hidden states. In order to introduce some flexibility, there are two parameters that introduce noise into the dynamics and the emission distribution. These parameters are set to best describe individual uncertainty, as inferred from Cognitive Tomography parameters (for more details see (Török et al., 2022). As a result of this parameterization, the Ground Truth model is capable of capturing the stimulus sequence perfectly but lacks the flexibility to capture individual differences between participants. The predictive performance of this model shows the extent to which participants were able to acquire the underlying stimulus sequence.

#### Cognitive Tomography.

This model was formalised as an Infinite Hidden Markov Model (iHMM), which is a nonparametric extension of the original HMM (Van Gael et al., 2008). In an iHMM the number of latent states (*s*_1_, *s*_2_, …, *s*_*n*_) is not fixed but it is learned from the data along with the continuous parameters of the HMM (the transition and emission matrices). In practice, a Bayesian estimate of the posterior over plausible latent states and parameters is obtained by collecting samples from the posterior. For this, a modified version of the beam sampler is defined (Török et al., 2022; Van Gael et al., 2008). Predictive probabilities are estimated by a Monte Carlo integral over samples. In contrast with the Ground Truth model, this model allows a hypothetically infinite number of hidden states, which enables this model to capture incomplete task representations, or faulty beliefs regarding the task structure: ∑_*s*_*t*_,*s*_*t*+1__ *p*(*y*_*t*+1_|*s*_*t*+1_) · *p*(*s*_*t*+1_|*s*_*t*_) · *p*(*s*_*t*_|*y*_1_, *y*_2_, … *y*_*t*_). Since the iHMM is an extension of the Markov and the Hidden Markov model, Cognitive Tomography is able to capture the dynamics of both the Ground Truth and the Markov models. Due to its flexibility, Cognitive Tomography can also capture highly complex sequence dynamics and therefore behaviors which can’t be described by previous simpler models.

#### Inference.

We use a custom sampling method, described in (Török et al., 2022). Briefly, we use a mixture of steps from the Hamiltonian Monte Carlo and the Gibbs sampling methods. Markov Chains use 1600 Gibbs-sampling steps (called slice sampling; Van Gael et al., 2008) and 30 Hamiltonian Monte Carlo are embedded in every single Gibbs step (for more details see Török et al. (2022)). To calculate predictive probabilities of upcoming stimuli we used four independently and randomly initialized chains, such that the last 30 samples were used from each chain to calculate predictive probabilities.

#### Response Model.

The generative model of response times relies on LATER (Linear Approach to Threshold with Ergodic Rate) model, which links subjective probabilities of upcoming stimuli with response times. A residual stochasticity of response times is describe by a reciprocal normal distribution (*r*_*n*_ ∼ *Normal*(*μ*, *σ*)). The LATER model uses the predictive probabilities (*p*_*n*_) established by the internal model for each observation: *RT*_*n*_ = $\frac{\theta_{0}-\text{log}\left(p_{n}\right)}{r_{n}}$, where *μ*, *σ*, *θ*_0_ are parameters characterizing individual’s response time model that were fitted for individuals and experimental sessions.

## RESULTS

In order to gain insight into the computations underlying transfer learning, first, we need to train participants to learn a task distinct from earlier experiences and identify the acquisition of new knowledge (i.e., tracing individual learning curves); second, we need to identify how the acquired knowledge can be exploited when a new but related task is introduced. Critically, learning is always a highly individual process: Learned representations are specific to and vary across individuals, even if subjects are exposed to a well-controlled environment during learning (Török et al., 2022; Zhao et al., 2023). As a consequence of strong across-individual variation of learned representations, averaging hinders the understanding of how learned knowledge is specifically harnessed in the updated setting. In order to respect this individual nature of learning, we measured the evolution of the internal representation during learning in a subject-by-subject manner. We used Cognitive Tomography (Houlsby et al., 2013; Török et al., 2022), a state-of-the-art computational tool to infer subjective internal representations from behavior. To study transfer learning, we assume that when individuals are exposed to a novel task, they rely on an inductive bias, which reflects their initial assumptions about task contingencies. Learning comprises two components: 1, By integrating evidence and the inductive bias, participants learn a model specific to the actual task; 2, Participants update their inductive biases such that in a novel situation, learning can exploit updated expectations. To identify hallmarks of transfer learning, we need to define a task i) for which we are able to reliably identify the inductive bias that participants maintain, and ii) requires participants to acquire a new internal model, which substantially differs from the previously maintained inductive bias.

To satisfy the above-described criteria, we need behavioral measurements which are informative about the internal models. When human participants are exposed to a stimulus sequence that instructs fast motor reactions in each trial, response times (RTs) are informative regarding the expectations of the upcoming stimuli (Janacsek & Nemeth, 2013; Kóbor et al., 2017; Noorani & Carpenter, 2016). We associate these expectations with the predictive probabilities of a probabilistic model. These subject-specific probabilistic models are identified with the subjective internal models participants build during exposure to the sequence.

### Internal Models Inferred From Response Times

**Figure 1.**
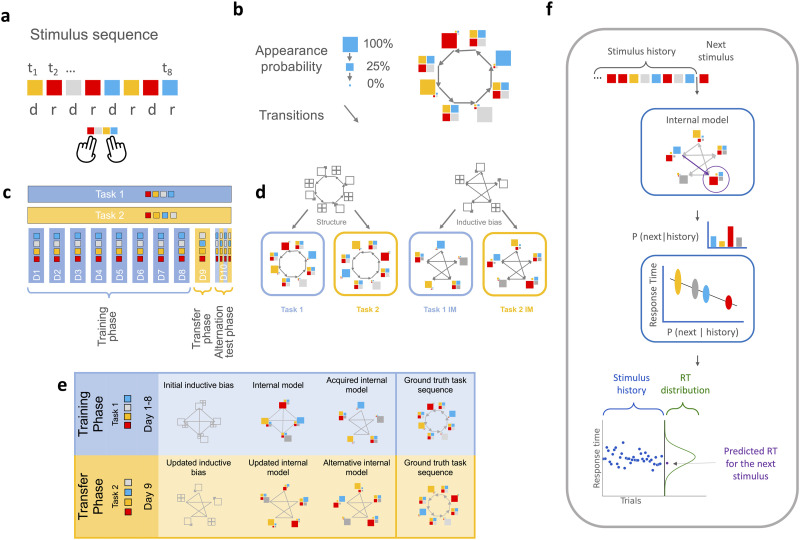
**Experimental paradigm and subjective internal representations**. **a**, An example segment of the stimulus sequence. Different colours indicate different stimuli. In a trial, one of four possible stimuli is presented, each associated with a different button that participants are required to press in order to progress the sequence. The eight-element sequence is repeated 10 times in a block. A training session consists of 25 blocks. In the eight-element sequence, random (r) and deterministic (d) elements are alternated. **b**, Representation of the probabilistic model governing the generation of the sequence. Progressing a sequence corresponds to transitions between states (black arrows). A state is characterized by the appearance probability of the four possible stimuli. The largest squares represent the deterministic states (100% appearance probability assigned to a specific stimuli, while the other 3 stimuli are associated with 0% appearance probability, represented by the smallest squares), states with four equal sized squares represent random trials (25% appearance probability associated with each stimuli). **c**, Training schedule. During the Training phase, participants are exposed for eight days (D1–D8) to a sequence governed by a given rule (blue shading, characterized by the red-yellow-gray-blue deterministic component, referred to as Task 1). During the transition phase on Day 9 of the experiment (D9) a new rule (corresponding to the red-yellow-gray-blue deterministic component, referred as Task 2) is introduced (yellow shading) that is a permutation of the rule governing the Training phase. In the Alternation phase on Day 10 of the experiment (D10) participants are exposed to both tasks, such that five-block-long sequences of the two tasks are alternated without cueing the transitions between tasks. **d**, Learning the stimulus sequence is hypothesized to occur on two levels: Learning the specific parameters that characterize each rule, and learning the underlying structure. The hierarchical nature of the task (left panel) enables us to test for these two-level computations. Under the general task structure, there are multiple possible tasks, governed by different rules. Out of the possible tasks corresponding to the structure we use two, one governing the Training phase, the other governing the Transfer phase (left panel, bottom row). Accordingly, we expect our participants to learn distinct internal models for the two rules albeit the internal models can be imperfect representations of the actual task (right panel). In the case of imperfect learning, the structure that can generalize across tasks corresponds to the number of states, transitions, and appearance probabilities characterizing the imperfect internal model. **e**, Schematics of the hypothesized acquisition of the new internal models. Top row: During the Training phase (D1–D8), learning starts with an initial inductive bias (Markovian in our case). As the training progresses, a new internal model develops, which throughout several days of training, becomes more similar to the real task structure. Bottom row: If learning entails the update of initial inductive biases, learning in the Transfer phase (D9) starts with the updated inductive bias, which supports the expedited development of a new internal model. **f**, Schematics of Cognitive Tomography. Based on stimulus history and the internal model (also inferred from RTs), appearance probabilities are calculated for the four stimuli at each trial. Given the individual RT parameters and the subjective probability of the upcoming stimuli, RTs are predicted subject-by-subject at each trial. Top box: Internal model. Colored squares indicate the probability with which any stimulus might appear in the given state, with square sizes being proportional to the appearance probabilities. Purple arrow shows momentary state transition, purple circle denotes the next state. The bar plot between the boxes shows subjective predictive probabilities for the upcoming stimulus. Middle box: Behavioral model (LATER), which relates subjective predictive probabilities to RTs. Higher subjective predictive probability will result in lower predicted RT and lower standard deviation. Bottom plot: Blue dots show RT history, the normal distribution shows the distribution of predicted RT upon the presentation of the next stimulus, and purple dot shows the RT sampled from the RT distribution.

We used the Alternating Serial Response Times (ASRT) task. In the ASRT paradigm participants are trained on a visual stimulus sequence of four stimuli in which the sequence alternates between random and deterministic elements. Participants (*n* = 25, see also Methods) are exposed to elements of the alternating sequence and are instructed to respond by pressing buttons associated with the identity of the observed stimulus (Figure 1a). The sequence progresses once a button has been pressed. The deterministic elements of the sequence are organized into a recurring 4-element-long sequence, which is thus interleaved with four randomly occurring stimuli (stimuli are sampled from a uniform distribution) (Figure 1b). While there is no direct incentive to learn the regularities of the sequence, participants are expected to implicitly learn the hidden sequence from observations (Farkas et al., 2024; Howard & Howard, 1997; Szegedi-Hallgató et al., 2019).

To assess the signatures of transfer learning in the ASRT task, we need to consider how inductive biases are updated through extended exposure to the task. For this, initial biases for sequential stimuli need to be identified. Humans’ ability to learn implicit sequential structure of stimuli has been demonstrated by a vast number of studies (Acuña & Schrater, 2010; Howard & Howard, 1997; Janacsek & Nemeth, 2013; Kóbor et al., 2017; Malassis et al., 2018; Newport & Aslin, 2004; Schulz et al., 2020). Learning dependencies of adjacent events is a skill exploited under several natural challenges, such as language acquisition, when learning and executing motor tasks, or when processing or producing music (Janacsek & Nemeth, 2013; Kóbor et al., 2017; Newport & Aslin, 2004). In contrast, learning non-adjacent dependencies appears to be more challenging to humans (Malassis et al., 2018; Newport & Aslin, 2004), which suggests that humans in general maintain priors for structures which are biased towards first-order dependencies. In line with these observations, previous research has shown that in an ASRT task initially the internal models of participants were dominated by a first-order Markovian structure (Török et al., 2022). In summary, the appeal of the ASRT task to study transfer learning is manifold. First, sequence prediction is a ubiquitous task. Second, the structure of the ASRT task is highly different from the natural dynamics of the environment, and a long learning process takes place upon extended exposure. Third, the initial assumptions (the inductive bias) of participants has been previously characterized as an inductive bias that is relatively homogeneous over the participants. Next, the ASRT provides a rich dataset as reaction time measurement is obtained for long sequences (80 key presses in 20 blocks in a given session). Finally, the high-level structure of the task and its potential contribution to transfer can be identified with computational methods.

Our approach aims at understanding whether a generalized structure representation is created by abstracting the task structure from the particular sequence or increased performance on the task merely reflects learning the stimulus sequence. While the latter option claims that participants can acquire only sequence-specific knowledge, the previous implies that participants are able to update their inductive biases and utilize the learned knowledge when facing similar but related tasks. To investigate this question, we apply a training schedule in which participants are trained for eight days (Training phase) using a stimulus sequence governed by one particular rule for the four stimuli in the deterministic component of the stimulus (Task 1), then on the ninth day (Transfer phase) we introduce a new rule to the deterministic component (Task 2) but retaining the overall task structure (Figure 1c, 1d). Finally, on the tenth day of the experiment, participants are exposed to the first and the second rule in an alternating manner, switching after every fifth block of trials (on Day 10 the session consists of 20 blocks of trials, see also Supplementary Material: Model Training and Evaluation). This alternation allows us to investigate the parallel maintenance of the two sequences: If the Training phase internal model is overwritten during the Transfer phase performance deteriorates on previously learned rule. If instead a new, separate model is learned during the Transfer phase, the two internal models are expected to be used in an alternating manner following the switches in stimulus sequences.

To capture the shifts in the internal models from the initial Markovian inductive bias towards a more complex internal model (Figure 1e), we need a framework that is able to flexibly discover internal models from a wider class of possible models. We use Cognitive Tomography to infer individualized internal models and to track individual learning curves. We use solely the sequence of response times of individuals to infer their internal model. Cognitive Tomography (CT) has two model components: The first infers individual internal models from response times, the second predicts reaction times to each stimulus based on the predictive probabilities of the internal models (Figure 1f). The internal model is formalized as a non-parametric version of the Hidden Markov Model called the Infinite Hidden Markov model (iHMM). The iHMM learns hidden stochastic dynamics over the state of the process and relates observations to these hidden states. Both the transitions between states and the observations come from probability distributions. Response time then depends on the probability of the upcoming stimulus, intuitively, a more expected stimulus is associated with a smaller response time. More formally, we use the Linear Ascend to Threshold with Ergodic Rate (LATER) (Carpenter & Williams, 1995) model (Török et al., 2022). For each participant and each day, we estimate the internal models separately. This approach lets us capture the day-by-day development of participants’ internal models. The goodness of inferred internal models is evaluated through their predictive performance, that is the variance explained (Pearson’s *r*^2^) in held-out RTs (Supplementary Material: Model Training and Evaluation). Note, that *r*^2^ is calculated on a trial-by-trial basis, instead of reporting trial-type averages. While a trigram model with 64 (4^3^) trial type results in close to 0.8 *r*^2^ values (Török et al., 2022), the trial-by-trial explained variance is lower.

**Figure 2.**
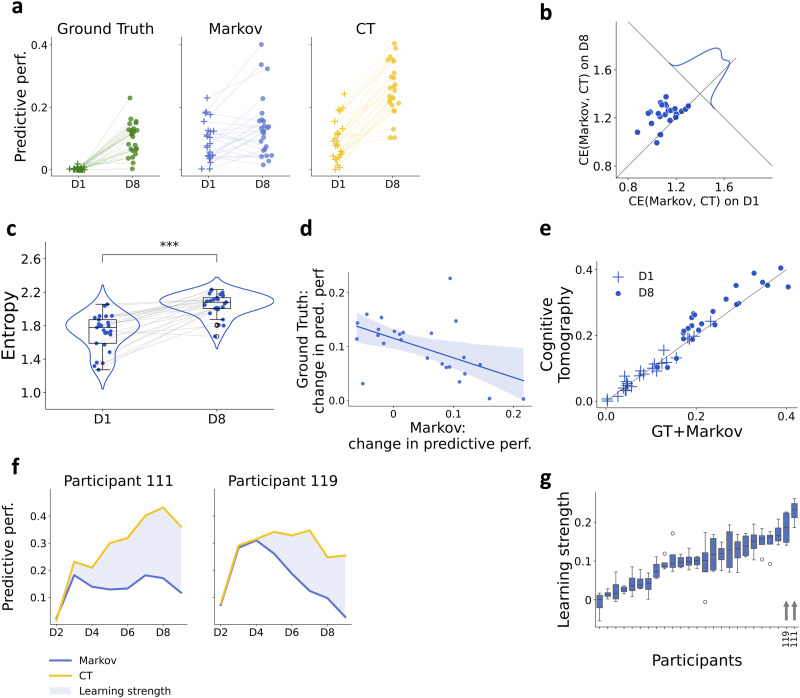
**Characterization of learning in the Training phase**. **a**, Predictive performance of the Ground Truth, Markov, and that of the Cognitive Tomography on the first and eighth days of training (D1 and D8, respectively). Markers indicate the predictive performance of individual participants. Initial overlap between the CT’s and Markov’s performance indicates that on the first day of training performance is dominated by a structure that is also captured by the Markov model. On D8 the behavior is better captured by the Cognitive Tomography than by the Markov for the majority of the participants, while the Ground Truth model also accounts for a larger amount of variance in the behavior, as opposed to the first day of training. **b**, Cross-entropy of Markov and CT models on D1 and D8. Dots show individual participants. Population level deviations from identity line show by a distribution plot. **c**, Latent state entropy on D1 and D8. Individual performances (dots) matched by a gray line across days. **d**, Change in the predictive performance of the Markov and Ground Truth model from D1 to D8. Dots show individual participants, blue shading shows the 95% confidence interval. **e**, Predictive performance of CT is proportional to the sum of variance explained by the Ground Truth and the Markov models. Markers indicate individual participants. On Day 1 (crosses) the explained variance is low, while by Day 8 (dots) predictive performance of CT reaches 0.4 for some participants. **f**, Evolution of the predictive performances of the CT and Markov models in the Training phase for two example participants. CT shows a consistent increase in predictive performance even with an eventual dramatic drop in the contribution of the Markov model. Colors of models as on panel a. Learning strength is the component of the internal model that is not explained by the Markov model (blue shading). **g**, Learning strength (averaged on D5–D8) across participants. Arrows indicate the example participants shown on c. Horizontal black lines represent the medians, boxes indicate interquartile ranges, and whiskers refer to 1.5 interquartile ranges below and above the first and third quartiles, respectively.

To characterize the initial biases and learning that unfolds after several day-long exposure to the ASRT task, we tracked the predictive performance of the CT across the eight days of the Training phase. Extended exposure to the stimulus sequence coincided with a systematic decrease in response times and an opposite trend only appeared upon the introduction of the new sequence on Day 9 (Supplementary Figure S1b, mean RT on D1 = 367.871, std = 32.8; mean RT on D8 = 281.427; std = 18.491, mean RT on D9 = 294.577, std 15.561). CT provides a tool to flexibly characterize the internal models of individual participants, but does not assess individuals against a learning objective. As a consequence, to assess learning performance we compare the CT-inferred internal model to a baseline. Along with the CT model, we also assessed the predictive performance of the Markov model, assumed to characterize the initial inductive bias, and the Ground Truth model, reflecting the actual structure of the stimulus sequence (Figure 2a). The Markov model substantially differs from the Hidden Markov Model assumed by the CT. The Markov model completely lacks internal states, which prevents it from having memory beyond the immediately preceding experience. Without internal states, the Markov model can only account for highly impoverished dynamics in the sequence of observations: Direct transitions between stimuli. The Markov and Ground Truth models allow us to interpret the properties of the internal models inferred by Cognitive Tomography. Since the Markov model only accounts for immediate temporal dependencies, while the Ground Truth model relies on temporal dependencies in which the first-order statistics is not relevant, these two models account for independent sources of variance. The variances thus are additive, and the variance explained jointly by the Markov and Ground Truth models closely matches the predictive performance of the Cognitive Tomography for each participant (Figure 2e). This observation let us break down the variance explained by Cognitive Tomography into meaningful components: The initial inductive bias and the learning strength. Since the predictive performance of the Cognitive Tomography and the Markov model are about the same level for each participant at the first days of training (Figure 2a), we identify the initial inductive bias with a Markov model. To quantify the level of match between the internal model identified by CT and the Markov model at the beginning and at the end of the Training phase, we calculated the cross-entropy between the Markov and CT models and found a consistent increase from D1 to D8 (Figure 2b, two tailed, paired *t*-test, *t*-value = −5.729, *p*-value < 0.001***, df = 24). Extended exposure to the ASRT task contributed to changes in the complexity of the inferred internal models of individuals (Supplementary Figure S1a). We assessed changes in internal model complexity by evaluating the latent variable structure of the inferred internal models. For this, we calculated the entropy of the latent states. The entropy measures the information associated with identifying a particular latent state of the internal model. This is a more intuitive measure than the number of latent states as the occupancies of the latents can be highly inhomogeneous, including extreme cases that a single state is always occupied with a number of unvisited states. The entropy was computed by calculating the distribution of latent state occupancies when data was synthesized from the internal model. Latent state entropy revealed a shift to more complex models by the end of Training phase (Figure 2c, two tailed, paired *t*-test, *t*-value = −7.058, *p*-value < 0.001***, df = 24). The predictive performance of the Ground Truth model captures how much the behavior reflects the actual stimulus sequence. Accordingly, the Ground Truth model cannot account for participant responses on D1 but the predictive performance increases considerably by D8. Variance across individuals in both the Markov and the Ground Truth model are considerable: While there is consistent increase across the population in the Ground Truth model, albeit with different intensities, there are both increases and decreases at the individual level in the Markov model (Figure 2a). We hypothesized that these changes are not independent: Those participants who acquire the Ground Truth model are more likely to rely on the Markov model later in the Training phase. Indeed, we found significant negative correlation between the changes in Markov and Ground Truth models from D1 to D8 (Figure 2d, *r* = −0.527, *p*-value = 0.007**). To gain insight into the internal model captured by CT, we investigated how much variance in response times CT accounts for and how much the Markov and Ground Truth models account for. We found that the joint predictive performance of Markov and Ground Truth models closely matches the predictive performance of the CT model both at the beginning and at the end of the Training phase (Figure 2e, *r* = 0.953, *p*-value < 0.001***). The increased amount of explainable variance on D8 compared to D1 is mainly accounted for by the acquired stimulus statistics (Figure 2a, 2d). Since the advantage of CT over the Markov model reflects the knowledge acquired about the task, we define the deviation of the predictive performance of the CT model from the Markov model as the learning strength of a participant (Figure 2f, see learning curves for all participants in Supplementary Figure S2, see also Supplementary Material: Measures for the Analyses). Thus, learning strength is a measure that characterizes the component of the predictive power of the CT, which goes beyond the Markov model. Learning strength reveals large heterogeneity of the pool of participants: Some showing negligible learning, while a larger population displays significant learning, albeit with varying levels of success during the Training phase (Figure 2g).

### Learning During the Training Phase Predicts the Success of Learning in the Transfer Phase

**Figure 3.**
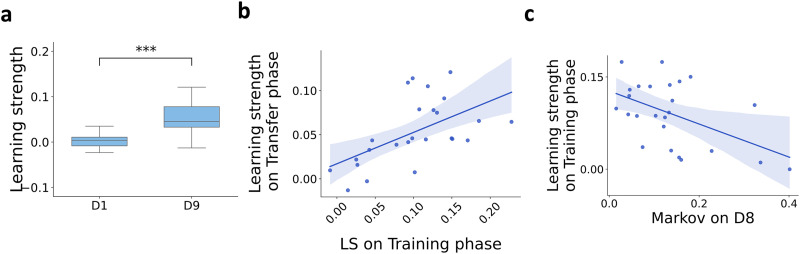
**Characterization of learning in the Transfer phase**. **a**, Learning strength on the first day of the Training and Transfer phases (D1 and D9, respectively). While learning strength on the first day of the Training phase was close to zero, increased performance on the first day of the Transfer phase is apparent. Horizontal black lines represent the medians, boxes indicate interquartile ranges, and whiskers refer to 1.5 interquartile ranges below and above the first and third quartiles, respectively. **b**, Learning strength on Task 1 (averaged across D5–D8) predicts learning strength on Task 2. Blue shading shows the 95% confidence interval. **c**, The Markovian structure is less dominant in the internal model of those participants who show greater learning strength. Dots show individual participants, blue shading shows 95% confidence interval.

Transfer learning is expected to facilitate learning a new task if its structure is congruent with earlier data. In terms of the ASRT task, transfer learning is expected to speed up learning of a new task if the ASRT task alternating structure of deterministic and random components is retained in a new Task but the deterministic pattern of Task 1 is replaced with a new one in Task 2 (Figure 1d). To test this, in the Transfer phase the deterministic components of the sequence were permuted, and participants were exposed to this updated sequence on Day 9 of the experiment. While the learning strength on Day 1 didn’t differ from zero (mean = 0.003, std = 0.014; one-tailed *t*-test, *t*-value = 0.181, *p*-value = 0.930, df = 24) there was a prominent difference between the predictive performance of CT and Markov models on Day 9 (mean = 0.053, std = 0.037; one-tailed *t*-test, *t*-value = 7.175, *p*-value < 0.001***, df = 24) and difference of learning strength across the days was highly significant (one-tailed, paired *t*-test, *t*-value = −6.686, *p*-value < 0.001***, df = 24) (Figure 3a). Higher learning strength on Day 9 and predictive power of learning strength between Training and Transfer phases are good indications that initial dominance of the Markov model does not return upon exposure to a new sequence. Successful transfer learning requires that upon the introduction of a new task, learning is more efficient as a result of updated inductive biases. Indeed, increased performance on D9 is consistent with this requirement. Furthermore, if the accelerated learning of the new rule on D9 emerges from the knowledge acquired during the previous eight days, varying performance on Task 1 should be reflected in individual performance level on Task 2 as well. Accordingly, performance on Task 1 predicts performance on Task 2 (*r* = 0.559, *p*-value = 0.004**) (Figure 3b). Consistent with this, we found that learning strength of participants predicted the magnitude of increase in response times between the end of the Training phase and the Transfer phase (Supplementary Figure S1c, *r* = 0.508, *p*-value = 0.010*). In contrast, the magnitude of response time reduction during the Training phase did not predict the increase between the end of the Training phase and the Transfer phase (Supplementary Figure S1d, *r* = 0.059, *p*-value = 0.779). Higher learning strength on the first day of exposure to the new task than on the first day of the initial exposure to the first task is a necessary condition for efficient transfer, but this analysis does not exclude the possibility of simply ignoring the new context while keeping the internal model acquired during the Training phase. Importantly, not only the learning strength on D8 predicts the learning strength on D9 but the contribution of the Markovian structure, which was identified with the initial inductive bias, too is correlated with acquiring a new model during the Training phase, as quantified by the learning strength on D8. A strong negative correlation is identified between the predictive performance of the Markov model on D8 and the learning strength on D8 (*r* = −0.500, *p*-value = 0.011*) (Figure 3c).

**Figure 4.**
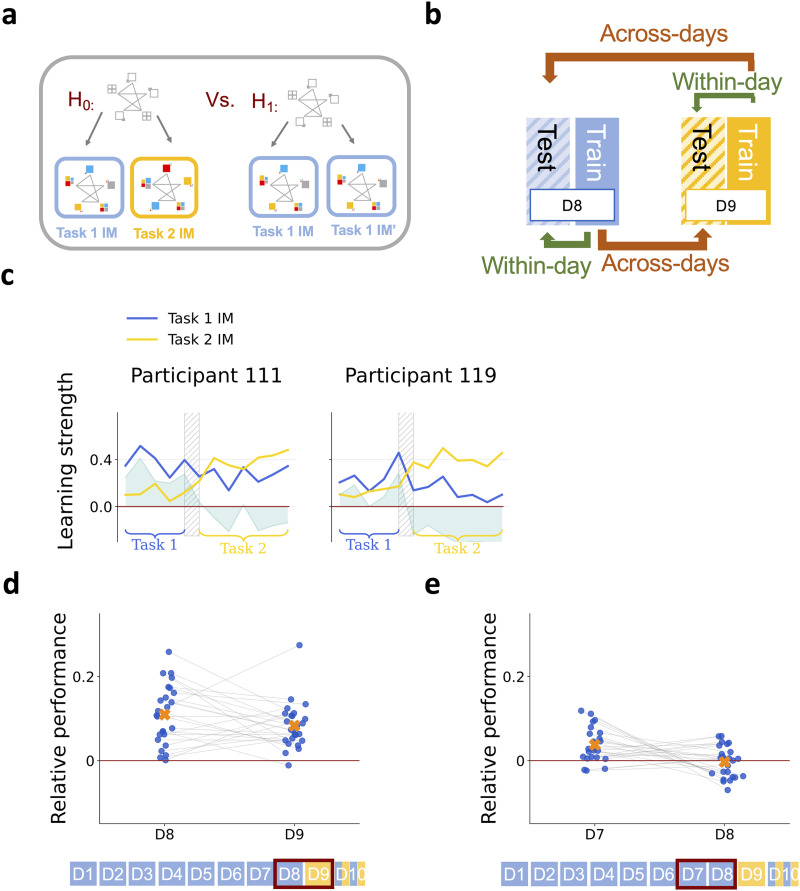
**Acquisition of a distinct internal model for Task 2 in the Transfer phase**. **a**, Alternative hypotheses for the enhanced performance in the Transfer phase (D9). H_0_: Emergence of a Task 2-specific new internal model explains the enhanced predictive performance. H_1_: The internal model used under Task 2 is a reflection of the proliferation of the Task 1 internal model. **b**, Schematics of inference and testing of internal models. CT is trained for each participant on D8 RTs and D9 RTs separately, then each trained model is tested on the training day (within-day, green arrows) and on the other day (across-days, brown arrows). **c**, Switching internal models for two example participants. Comparison between the within-day and across-days performance of the inferred internal models enables us to evaluate the level of dissimilarity of the two internal models. Crossover of predictive performances of D8 (blue line) and D9 (yellow line) internal models shows the switch between internal models when the task changes between D8 and D9. The extent of divergence in the two internal models reflects the dissimilar predictive power (green shading) of the two internal models on Task 1 (D8) and on Task 2 (D9). **d**, Relative performance of within-day and across-days model predictions on Day 8 and Day 9 for the pool of participants (dots). Higher values reflect stronger divergence between the internal models inferred on separate days. Orange crosses show the group mean. **e**, Same as d, but for two consecutive days during the Training phase (Day 7, and Day 8).

To test whether the increased learning strength on Day 9, while exposed to Task 2, corresponds to the recruitment of a new internal model or a proliferation of the previously acquired model (Figure 4a), we trained separate models for the last day of Task 1 and for the first day of Task 2 and contrasted the predictive performance of these models. Importantly, retaining the Task 1 internal model would mean that across-days predictions are equally strong as within-day predictions. We inferred internal models on the second half of the blocks of trials and made predictions on the early blocks of trials on the same day (within-day prediction) as well as early blocks of trials on different days (across-days prediction, Figure 4b, see also Supplementary Material: Measures for the Analyses). While proliferation of the internal model in the Transfer phase would imply strong across-days predictive performance, recruitment of a new internal model would be expected to display a crossover in the two internal models’ predictive performance: Within-day predictions are expected to be strong, as opposed to a drop in predictive performance across-days (Figure 4c). We directly tested this crossover by assessing the difference between within-day and across-days predictive performances of the Task 1 and Task 2 internal models (Figure 4d). The relative performance of the within-day model was significantly above zero both on Day 8 and on Day 9 for the pool of participants (two-tailed *t*-test, *t*-value = 7.713, *p*-value < 0.001***; *t*-value = 7.500, *p*-value < 0.001***, df = 24, for Day 8 and Day 9 respectively). There was considerable variance across participants, in line with earlier observations about the diversity of learning strategies. To exclude the possibility that the dissimilarity of internal models reflects a non-specific deterioration of predictive performance of an internal model between days, not a change between tasks, we performed the same dissimilarity test on Day 7 and Day 8 of the Training phase. Relative performances were significantly higher for the Day 8 and Day 9 comparisons than for the Day 7 and Day 8 comparisons (anova *f*-value = 23.937, *p*-value < 0.001*** df = 24, to compare groupwise means we used the Tukey’s Pairwise Group Comparisons, where all the relevant comparisons were highly significant, p-value < 0.001**). These findings show that the majority of participants were able to develop a new internal model when exposed to the new stimulus sequence, which could explain their behaviour specifically during Task 2 blocks of trials but not during Task 1 blocks.

**Figure 5.**
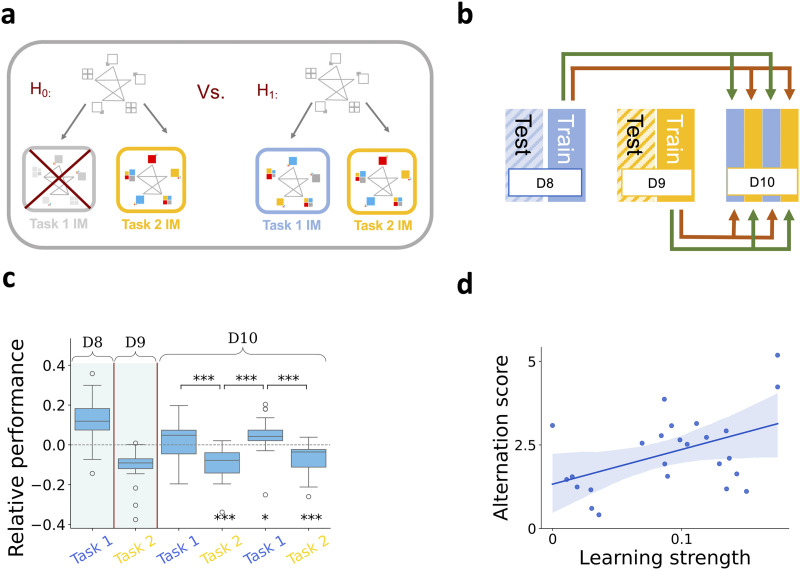
**Parallel maintenance of two internal models**. **a**, Alternative hypotheses for the diverging performance of the two internal models on D8 and D9. H_0_: The internal model for Task 1 was abandoned and replaced by the internal model for Task 2. H_1_: Training on different stimulus governing rules entails the development of an inventory and parallel maintenance of two internal models. **b**, Schematics of inference and testing of parallel maintenance of two internal models on the alternating blocks of trials in the alternation phase (D10, stimulus governing rules alternates between Task 1 and Task 2 in five-block batches, colors as in earlier figures). CT is trained for each participant on D8 RTs and D9 RTs separately, then each trained model is tested on the corresponding (green arrows) and non-corresponding (brown arrows) task sequences of D10. **c**, Participants alternate between the two internal models according to the stimuli sequence. Relative performance is measured as a difference in the variance explained by the Task 1 internal model and Task 2 internal model. Advantage of the corresponding internal model shown by positive values on Task 1 blocks, and negative values on Task 2 blocks. The first two boxes show the within-day relative performances of D8 and D9 internal models, establishing a baseline for the expected performance measures in case of model switch. The alternating rules of the last four blocks show participants’ ability to switch between internal models according to the alternating stimuli sequence on D10. **d**, Participant-by-participant (dots) assessment of the ability to alternate between two internal models as a function of learning strength during the Training phase (measured on D5–D8). Blue shading shows the 95% confidence interval.

Fast recruitment of a novel internal model during the Transfer phase indicates that learning is supported by effective inductive biases. However, the question remains open if this high level of learning enables humans to extend the initial internal model to an inventory by parallel maintaining internal models that are adequate for the Training and Transfer phases separately. If the training process entails transfer learning, the old and the new internal model coexist, otherwise, the old model is abandoned (Figure 5a). To test if parallel maintenance of internal models occurs, we extended the experiment by an additional session: On Day 10, in the Alternation phase, rules governing Task 1 and Task 2 were alternated in every five blocks of trials, without cueing the transitions (Figure 1c). If the two internal models are maintained parallel, participants are expected to alternate between them according to the stimulus governing rule. To test the parallel maintenance of the two models, we compared the predictive performance of the Task 1 and Task 2 internal models on the alternating blocks of Day 10 (Figure 5b). Participants’ ability to alternate between internal models is reflected by the relative advantage of the corresponding internal model (within-task prediction) compared to the other one (across-tasks prediction; Figure 5c). The ability to alternate between the two internal models varied across participants, and this ability was, again, related to the ability to acquire new knowledge during Task 1 exposure. High learning strength on Task 1 predicted the ability to alternate between internal models according to the alternating stimuli (r = 0.473, p-value = 0.017*, Figure 5d).

### Subjective Belief Transferred Across Tasks

**Figure 6.**
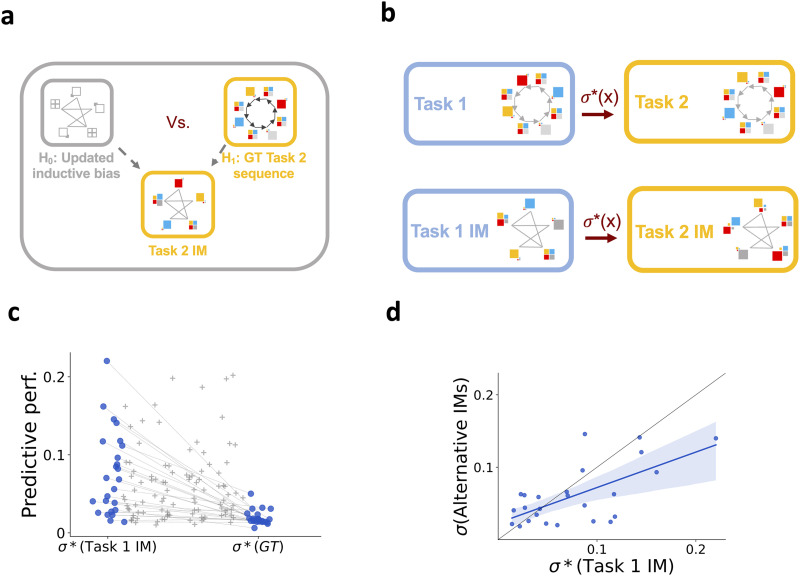
**Subjective belief transferred across tasks**. **a**, Alternative hypotheses for characterizing the Task 2 internal model. H_0_: The Task 2 internal model is derived through the structure of the internal model acquired during the Training phase. The structure of the Task 1 internal model determines the structure of the internal model of Task 2. H_1_: The Task 2 internal model is derived from the Ground Truth sequence of Task 2. The assumption behind this hypothesis is that the gradual alignment of the internal representation of individuals with the Ground Truth Task 1 model during the Training phase enables participants to pick up the matching-structure Ground Truth model of Day 9 sequences. **b**, The Task 2 Ground Truth model can be obtained from the Task 1 Ground Truth model though a specific permutation of the possible observations associated with the states of the Task 1 model (normative permutation, σ*). Assuming that participants have only access to their subjective belief about the ‘mechanics’ of the task, transfer of the acquired knowledge about the task structure entails applying the same normative permutation on the subjective internal model as the one applied to the Ground Truth model. Constructing a ‘normative’ internal model for Task 2 by this permutation enables us to evaluate the contribution of the subjective task representation (developed during the Training phase) to the behavior on Task 2. **c**, Comparing the predictive performance of the normative permutation of the Task 1 internal model (σ*(Task 1 IM)) and that of the Ground Truth model. Dots show individual participants, grey crosses show alternative permutations of individual internal models. **d**, Predictive performance of the normative permutation (σ*) of the Task 1 internal model on Task 2 response times against the predictive performance of alternative permutations of the Task 1 internal model. Black line indicates identity. Dots show the permutations for individual participants, blue line is linear regression of the data performances and shading is the 95% confidence interval.

After showing that there are distinct internal models maintained for Task 1 and Task 2, the remaining question is how we can characterize the transferred representations, i.e., the properties of the initially acquired internal model that are reused while learning in the Transfer phase. During the Training phase (Day 1–Day 8), the internal model can gradually be better characterized by the Ground Truth stimulus sequence (Figure 2a). One hypothesis of the way knowledge acquired during the Training phase can be identified in the Transfer phase is assuming that the Ground Truth model of Task 2 is tested in the Transfer phase: Since individuals show signatures of acquiring the Ground Truth structure by Day 8 (Figure 1a), the matching structure of Task 1 and Task 2 could immediately empower participants to effectively learn an internal model reflecting the properties of Task 2. (Figure 6a, H_1_). However, we observed less-than-perfect learning of the ground truth sequence in individuals, relying on a combination of the initial inductive bias and the Ground Truth model even after eight days of training. As a consequence, an alternative hypothesis is that it is the internal model corresponding to the subjective belief of the participant that is reused in the new task context (Figure 6a, H_0_). This internal model is referred to as subjective, since it’s highly diverse across participants, and is an imperfect representation of the task structure that incorporates the characteristics of the task that a participant was able to pick up. We hypothesize that during subjective transfer, we can characterize the internal model of Task 2 by applying the permutation matching the experimental manipulation to the Task 1 internal model (Figure 6b).

We contrasted these two hypotheses by calculating the predictive performance of two models: 1, The permutation of the Ground Truth model; 2, The permutation of the subjective internal model. We found that, the predictive power of the permuted subjective internal model was significantly higher compared to the predictive power of the permutation of the Ground Truth model on the Transfer phase (permuted Ground Truth model mean = 0.019, std = 0.009, permuted subjective internal model mean = 0.075, std = 0.053; two-tailed, paired *t*-test *p*-value < 0.001***, *t*-value = 5.446, df = 24) (Figure 6c). This indicates that the structure that characterizes the internal model participants acquire during the Training phase (Task 1) successfully generalizes to Task 2. Further, this transferable knowledge reflects more the characteristics of the internal model, they developed during the Training phase, than the characteristics of the Ground Truth task sequence during the Transfer phase.

We tested the specificity of this prediction by constructing alternative internal models: Instead of the ‘normative’ permutation motivated by the actual experimental manipulation, we constructed alternative internal models corresponding to the four alternative permutations (see Supplementary Material: Measures for the Analyses) of the original deterministic sequence. We compared the predictive performances of the normative permutation and those of alternative permutations (Figure 6d). Predictive performance of the normative permutation was significantly higher than that of the alternative permutations (*t*-test *t*-value = 5.098, *p*-value < 0.001***, df = 3123). Thus, our results show that the structural characteristics of the internal model acquired during the Training phase are significantly contributing to the behavior of participants in the Transfer phase. This contribution is indirect: It is a transformation of the inferred subjective internal model that predicts responses in the Training phase, a signature of reusing structural knowledge in a new context.

## DISCUSSION

In this paper, our goal was to characterize the computations that facilitate transfer learning. Transfer learning requires the acquisition of a representation that is abstract enough to generalize to related but novel tasks and situations. We investigated the acquisition and exploitation of these task representations. For this, we analyzed behavioral responses in a task that is dissimilar enough from initial inductive biases so that participants needed to update their inductive biases. This enabled us to investigate specifically the contribution of that newly acquired inductive bias under a novel task. We showed that i) eight days of training is sufficient to update inductive biases and incorporate a structure that is distinct from initial priors; ii) updated inductive biases enable accelerated learning of tasks analogous to the original task; iii) training on a pair of tasks contributes to building an inventory of internal models, that is to maintain multiple models that can be recruited when circumstances necessitate their use; iv) transfer of implicit knowledge across tasks could be identified through the transformation of a subjective task representation.

Learning can be understood as a two-level computation: At a lower level, within a model family, parameters of the model need to be adjusted to reflect the properties of the environment; while on a higher-level, the right structure needs to be discovered (Fiser et al., 2010; Kemp et al., 2007; Wang, 2021). Earlier studies identified this problem as a form of Bayesian model learning and demonstrated that high-level learning can be accomplished on a rich variety of data such as object properties, causal structures or judgements (Braun et al., 2010; Gershman & Niv, 2010; Kemp & Tenenbaum, 2008, 2009; Lucas et al., 2014; Lucas & Griffiths, 2010; Zhao et al., 2023). Our work is aligned with this line of research: We assumed that initially both the structure and the parameters need to be learned by participants. Notably, we investigated a special form of such learning, learning about the sequential structure of stimuli and the structure-level knowledge was interpreted as an inductive bias for acquiring novel models of sequential stimuli. Earlier analysis has revealed that the initial inductive bias that participants rely on is heavily biased towards immediate dependencies, a simple structure that can be a parsimonious explanation in everyday circumstances and abandoning this inductive bias required extensive exposure (Török et al., 2022). In this Markovian structure transitions between observations are plagued by high uncertainty, rendering the behavior to display short time scale dependencies only (Éltető et al., 2022). Our current analysis has demonstrated that once the structure and parameters had been learned, the change in the task permitted the reuse of the learned structure. Indeed, in this new context those participants were more successful who displayed stronger signatures of learning in the Training phase and the structure of the internal model we inferred from responses was indicative of the model used in the Transfer phase.

In this paper, we argued that transfer learning capabilities of humans can be understood as the acquisition of the structure of the environment, and it is the learning of a higher-level structure that enables effective generalization and faster acquisition of new regularities. Structure learning has gained strong support in a wide array of cognitive phenomena (Heald, Wolpert, et al., 2023) such as classical conditioning in humans and animals (Courville et al., 2006; Gershman et al., 2010; Gershman & Niv, 2010) visual learning in humans (Orbán et al., 2008) inductive reasoning in humans (Kemp & Tenenbaum, 2009), sensorimotor learning in human (Braun et al., 2010; Heald et al., 2021; Tian et al., 2020). From a theoretical point of view, structure learning has been implicated as the basis of transfer learning (Hassabis et al., 2017; Wang, 2021). Our paper provides support to this idea, as we have shown that a structure learning account efficiently predicts reaction times in a transfer learning setting. However, our study does not provide a process model for transfer learning since CT only captures a snapshot of the internal representation individuals maintain.

Discovering the finer details of the principles of transfer was limited by a number of factors. First, the flexibility of CT comes with a limitation too: Stable inference of the iHMM from reaction times requires ten blocks of trials (Török et al., 2022). This can be somewhat reduced, still tracking more granular processes is limited by this property. Alternative probabilistic models with more constraints to characterize the internal model can be more effective than iHMM albeit less precise or flexible. Furthermore, using other behavioral modalities, such as gaze, might provide more information on the predictive probabilities underlying actions. Second, the population of participants displayed a high level of diversity in learning strategies in the Training phase, which differences were exacerbated in the Transfer phase. Next, during the Transfer phase of the experiment CT attempts to capture an internal model that is not stable because of potential variance in the time of abandoning the Task 1 model, as well as Task 2 model is being learned in the blocks used for inference. This volatility means that assuming a single internal model during the Transfer phase is just a coarse approximation, and predictions for the one internal model inferred by CT is more limited than in the Training phase. Nevertheless, the indication that a simple transformation of the internal model was predictive to the internal model used in the new context provides a promising indication that the two levels of learning can be identified in this setting.

With respect to the acquisition of a generalizable structure, earlier considerations highlighted that a diverse set of tasks can effectively contribute to the establishment of such a structure (Behrens et al., 2018; Harlow, 1949; Samborska et al., 2022; Tse et al., 2007; Whittington et al., 2022). In our paradigm, a single task was used to establish this generalizable structure. It could be a further line of investigation of how task diversification can promote more effective transfer. We identified successful switching between internal models when the rules of the sequence change. Tracking of the actual rule is tightly related to contextual learning (Heald, Lengyel, et al., 2023). When no cue is available for an observer to establish the actual rule, they need to learn a higher-level representation, which can be thought of as a context that establishes the actual task rules (Heald et al., 2021). The fact that a subset of participants could reliably switch between Task 1 and Task 2 internal models in the Alternation phase indicates that such internal representation of context has been established by these participants.

Transfer learning has been widely studied in the machine learning literature, which might provide insight into the underlying computations that enables the analogue mental process in primate cognition. In the machine learning literature approaches can be classified into two broad classes. A training objective-driven approach focuses on how efficient transfer can be achieved. These span methods such as engineering well-transferable weights, finding a suitably flexible model architecture (Duan et al., 2009; Galanti et al., 2022; Hong et al., 2016; Parisot et al., 2022; Yang et al., 2007; Zhuang et al., 2021), and this approach has been successfully applied to cognitive phenomena (Mark et al., 2020; Nelli et al., 2023). An alternative approach emphasizes the importance of learned representations to improve transfer learning, often relying on hierarchies and structures that could meaningfully shape meta-level task representations (Battaglia et al., 2018; Finn et al., 2017; Grant et al., 2018; Madarasz, 2022; Madarasz & Behrens, 2019, 2020; Ortega et al., 2019; Neal & Shahbaba, 2007; Srivastava & Salakhutdinov, 2013). This line of research is well aligned with our study presented here. However, a process model that would help understanding the acquisition and manipulating knowledge at the level of structures was not addressed. While such models exist in other domains (Kemp et al., 2007; Mark et al., 2020), identifying such a process model in our sequence learning setting is a subject of future research. Recently, compelling strides have been made in how the neural circuitry is contributing to these computations (Goudar et al., 2023; Whittington et al., 2020), which might establish a framework to a biological theory of transfer.

Transfer learning is considered to be a fundamental skill of humans and has been identified in studies on non-human primates as well (Harlow, 1949). Still, the computations underlying flexible transfer are not fully understood. An important insight is that a model of the environment is a critical component of an artificial agent that can reuse its knowledge in novel situations (Walker et al., 2023). Indeed, our work showed that an internal model of the task can be reused in novel situations. Thus, studying transfer in humans might provide guidance for building better and more human-like AI.

## AUTHOR CONTRIBUTIONS

A.S.: Conceptualization; Formal analysis; Methodology; Software; Visualization; Writing – original draft; Writing – review & editing. G.O.: Conceptualization; Funding acquisition; Methodology; Writing – original draft; Writing – review & editing. B.T.: Data curation; Methodology; Software. M.K.: Data Curation. K.J.: Data curation; Funding acquisition. D.N.: Data curation; Funding acquisition.

## FUNDING INFORMATION

This research was supported by the ANR grant awarded within the framework of the Inserm CPJ (D.N.), the National Brain Research Program by Hungarian Academy of Sciences (project NAP2022-I-1/2022) (D.N.); the Hungarian Ministry of Innovation and Technology NRDI Office within the framework of the Artificial Intelligence National Laboratory Program and by the project TKP2021-NKTA-62 (G.O.).

## ETHICS STATEMENT

Participants were required to provide written informed consent before enrollment of the experiment. Participants received course credits for taking part in the experiment. The study was approved by the United Ethical Review Committee for Research in Psychology (EPKEB) in Hungary (Approval number: 30/2012) and by the research ethics committee of Eötvös Loránd University, Budapest, Hungary. The study was conducted in accordance with the Declaration of Helsinki.

## DATA AND SCRIPT AVAILABILITY

Scripts for the model training: https://github.com/mzperix/asrt-beamsampling. Experimental data and scripts for the analyses and plots: https://github.com/CSNLWigner/cogtom_transfer_learning.

## Supplementary Material


