# Supplementary material for: Identifying Transfer Learning in the Reshaping of Inductive Biases
Source: Open Mind (Camb). 2024 Sep 15;8:1107–28. doi: 10.1162/opmi_a_00158 (PMC11410354; doi:10.1162/opmi_a_00158)
Supplement: Supplementary file 1 [file opmi-08-1107-s001.pdf]

Identifying Transfer Learning in the Reshaping of Inductive Biases
Supplementary Material

Anna Székely, Balázs Török, Mariann Kiss,
Karolina Janacsek, Dezső Németh, Gergő Orbán

Cognitive Tomography[1]

The infinite Hidden Markov Model (iHMM) is a non-parametric extension of the Hidden Markov Model, which features a flexible number of hidden states and thus permits the data to determine the model complexity. In practice, the iHMM assumes infinitely many hidden states of which a finite number is practically used. The parameters of the iHMM are conveniently mapped onto the parameters of the classical Hidden Markov Model [2], which we refer to as the transition and emission matrices, aligning with the conventional terms. Bayesian learning of model parameters requires the specification of priors for model parameters. These priors are hierarchical to accommodate the changing number of parameters for different numbers of latent states. We use the hierarchical prior defined in [3]. We extended the original implementation to accommodate a doubly Bayesian treatment (see below).

Below we first introduce the doubly Bayesian treatment, then we provide a detailed description of the iHMM model, then we introduce the response time model, and finally we describe learning of the iHMM model from response time data.

This model description is a shortened version of the one in the original publication [1].

Doubly Bayesian Model

Due to the uncertainty of the participants about the true model and actual state of the stimuli and to the uncertainty of the experimenter about the model maintained by participants and about the actual state of this internal model, the problem can be described as doubly Bayesian.

[1] Note that Cognitive Tomography is the same model used and described in [1]. Here we provide a general description of the modeling method, for more details, see [1]

We do Bayesian inference over an internal representation of individuals who themselves do Bayesian inference. Elements of the experimenter's model are introduced in following sections.

Prediction of response times can be described by the following algorithm:

1. We take posterior samples from the behavioural model which consists of parameters of the internal model and the response time model conditioned on data from ten consecutive blocks of trials, where:
   (a) all stimuli, and
   (b) response times (with incorrect trials', first five random trials' response times, and response times smaller than 180 msec in each block removed). According to the original formulation by [4], fast response times come from an alternative distribution. We cut off the fast response times (as in [5]) at the fixed 180 msec value. However, we did not fit the cut-off time parameter.

   are included.

2. For each of the 60 posterior model samples we compute predicted response times by:
   (a) filtering the belief over the latent state over the entire sequence
   (b) produce subjective probabilities for each trial
   (c) produce response time prediction (MAP estimate conditioned on the subjective probability and the response time parameters of the model sample)

   Then we marginalize (i.e. average) over the response time predictions of model samples.

3. We evaluate model performance by computing the r^2 explained variance measure of the predicted response times on the response times of the test dataset.

Note: since actual beliefs depend on past beliefs, one can think of the belief sequence as the path of a light-ray in a large dimensional fog (representing the state uncertainty). During inference, we have a noisy measurement of the light-ray in different points of time and we would like to reconstruct the best explanation of the observation sequence (response times) in terms of a hidden path. As for prediction, the model produces response time predictions for the entire stimulus sequence with no further feedback of response times (i.e. estimated internal beliefs are not updated based on what response time the participant produced on given trials).

Infinite Hidden Markov Model

The internal model of a participant is assumed to be a probabilistic model of the stimulus sequence, which is formalized as an iHMM. At trials t, observed stimulus, Y_t, is governed by a latent (not directly observable) state S_t. The latent state at trial t only depends on the latent state in the immediately preceding trial:

$$p(S_t|S_1, S_2, ..., S_{t-1}) = p(S_t|S_{t-1}).$$

Thus, the state S_{t-1} carries all information about the past for predicting the current state, S_t. Importantly, latent states can introduce an arbitrary-horizon dependencies between observed stimuli.

Transition between latent state i and j is governed by the transition matrix, π:

$$\pi_{i,j} = p(S_t = j|S_{t-1} = i)\ \forall t.$$

Potential observations at trial t are governed by the emission matrix, ϕ. For latent state i the probaiblity of stimulus k is

$$\phi_{i,k} = p(Y_t = k|S_t = i).$$

At any given trial, we assume the participant had estimated the parameters π and ϕ and uses these to infer the latent states from the sequence of observations. When predicting response times, we hold the internal model fixed within shorter time-scales of the task (e.g. a set of blocks, 10 blocks by default). The participant represents their belief about the current latent state of the system by a posterior distribution. This posterior is updated with observing a new stimulus such that using the actual parameters estimates $\hat{\pi}$ and $\hat{\phi}$:

$$\begin{aligned}\hat{s_t} := p(S_t = s_t|Y_1 = y_1, Y_2 = y_2, ..., y_t) \propto& \\ &\propto p(y_t|s_t)p(s_t|y_1, y_2, ..., y_{t-1}) \\ &= \sum_{s_{t-1}} \hat{\phi}_{s_t,y_t} p(s_t|s_{t-1})p(s_{t-1}|y_1, ..., y_{t-1}) \\ &= \sum_{s_{t-1}} \hat{\phi}_{s_t,y_t} \hat{\pi}_{s_{t-1},s_t} \hat{s}_{t-1}.\end{aligned}$$

For prediction of upcoming stimuli, stimuli from all trials were used (including initial random trials at the beginning of each block and stimuli in trials where participant committed an error). That is, even if response times were not considered when doing inference over the participant's internal model, the participant was assumed to update their beliefs about the latent states based on the presented stimulus.

Predictive probability for the upcoming stimulus was computed by marginalizing over the distribution of potential latent states (termed the latent state posterior):

$$
\begin{aligned}
p(y_{t+1}|y_1, y_2, ..., y_t) = & \\
&= \sum_{s_{t+1}} p(y_{t+1}|s_{t+1}) p(s_{t+1}|y_1, y_2, ..., y_t) \\
&= \sum_{s_{t+1}} \hat{\phi}_{s_{t+1} y_{t+1}} p(s_{t+1}|y_1, y_2, ..., y_t) \\
&= \sum_{s_t, s_{t+1}} \hat{\phi}_{s_{t+1} y_{t+1}} p(s_{t+1}|s_t) p(s_t|y_1, y_2, ..., y_t) \\
&= \sum_{s_t, s_{t+1}} \hat{\phi}_{s_{t+1} y_{t+1}} \hat{\pi}_{s_t, s_{t+1}} \hat{s}_t.
\end{aligned}
$$

Response Time Model

In order to connect the predictions of the internal model to measured behaviour, we need to employ a generative model of response times. In this generative model, response times are assumed to the upcoming stimulus was dependent of the predictive probability of that stimulus. The probability of response times was following the LATER model, originally developed for reaction times in saccadic eye movement experiments [4]:

$$
\begin{aligned}
r_n &\sim \text{Normal}(\mu, \sigma) \\
RT_n &= \frac{\theta_0 - \log(p_n)}{r_n}
\end{aligned}
$$

where p_n is the predictive probability of the upcoming stimulus (output of the internal model) and μ, σ, θ_0 are the parameters characterising an individual's response time model. These parameters jointly describe the mean and variance of the response times. Note that in our experiment these parameters comprise all idiosyncratic effects at hand, namely the

individual's state, their response times' sensitivity to subjective predicted probabilities, the effects of instruction influencing speed-accuracy trade-off.

Response time parameters are learned along with the parameters of the internal model. The priors of the response time parameters were

$$\tau_0 \sim \Gamma(1, 10)$$
$$\mu \sim \Gamma(1, 0.1)$$
$$\sigma \sim \Gamma(1, 0.01)$$

Learning

Trial-by-trial updates to the internal model were not calculated. Instead, internal models were estimated based on a fixed number of blocks of trials. For learning the internal model parameters the priors for the transition and emission matrices were

$$\hat{\pi}_i \sim \text{Dirichlet}(\alpha_0/K, ..., \alpha_0/K, \alpha_0/K \cdot \epsilon)$$
$$\hat{\phi} \sim \text{Dirichlet}(0.8, 0.8, 0.8, 0.8)$$

The hierarchical prior over the transition matrix was characterized by parameters $\alpha = 1.3$ and $\gamma = 3.8$ [3].

Learning the internal model constitutes the calculation of the parameter posterior. Note, that as there is uncertainty over the structure of a participant's internal model (most notably the number of latent states, denoted by K), a point estimate of the parameters is not sufficient. Instead, the doubly Bayesian approach requires the estimation of a posterior distribution over the parameters. Exact inference of the parameters of the iHMM is not possible, approximate inference is used instead. A particularly effective form of approximate inference for non-parametric Bayesian models, such as the iHMM is sampling. Sampling-based learning implies that instead of optimizing the parameter set of the model, stochastic samples from the posterior are systematically collected. Thus, the result of learning is a set of potential model parameters that faithfully represent the posterior distribution over the parameters. Bayesian learning dictates that predictions are calculated by integrating over the parameter posterior, we use the stochastic samples from the paramter posterior to calculate a Monte-Carlo integral.

We used a custom sampling method that mixes steps of a Hamiltonian Monte Carlo (HMC) and a Gibbs sampler which samples a slicing parameter (see [3]). In order to handle the infinitely many possible states, we use a modified version of this slice sampling method.

Instead of sampling the latent state sequence and make use of the slicing variable to constrain the set of used states to a finite set and sampling the latent sequence and the slicing variables in an alternating fashion, we did not sample latent state sequences, but estimated the subjective belief sequence over the latent states. In this latter case, the posterior belief was infinite dimensional and we used slicing to approximate this infinite-dimensional computation with a finite one. At each sampling step, we only looked at the latent state belief distribution's $1 - \epsilon$ support where ϵ was sampled from Uniform$(0.02, 0.2)$.

Four independently and randomly initialised Markov Chains were sampled with 1600 steps of the slice sampling (outer Gibbs-sampling chain) and 30 NUTS steps were taken in between slice sampling steps each time. Samples from the second half of each chain were used to check if estimates of response time parameter means and confidence intervals were identical. For prediction, last 60 unique samples were used from each chain because prediction performance saturated at this number of samples.

Model Training and Test

We train separate models for each participant on each day. This allows us to infer individual internal models, and their development day-by-day. For this, we split the block of RTs into training and test datasets. On the Training Phase (between Day 1 - Day 8) we split the 25 blocks of trials in each training session such that we used the second 10 blocks (11-20) to train the models and the first ten blocks (1-10) to test the models. On Day 9 we trained models on the last ten blocks (16-25), to capture the most developed internal model, that our participants are able to develop during the unique session of the Transfer Phase, and tested on the first fourteen blocks of the day (1-14) in order to capture the gradual development of the Task 2 internal model. On Day 10 we did not train models, but tested previously inferred internal models, the D8 internal model and D9 internal model (that in this case was trained on the last eight blocks of the D9 training session: blocks 18-25). We tested the two internal models on the latter four blocks of the identical block segments on D10. In practice, on Day 10 we tested the models on blocks 2-5 (Task 1), blocks 7-10 (Task 2), blocks 12-15 (Task 1), and on blocks 17-20 (Task 2). The models used for permutation were trained on D8 and D9 as detailed above, and tested on the first 22 block of D9, since no blocks needed to held out to avoid overlaps in the training and test data.

Model Evaluation

The goodness of inferred internal models is measured by their predictive power, i.e. the variance explained (Pearson's r^2) in the test block RTs by the predicted RTs. Correlation coefficient was computed for each participant and each block separately. For session-wise performance measures r^2s are averaged on the test blocks.

Model Validation

We performed a basic test of the goodness of the internal model inferred by CT by contrasting the magnitude and the distribution of response times against the empirically measured response times (Supplementary Figure S1e, S1f). Further, we checked the residuals of the CT predictions to assess their compatibility with the assumption about normally distributed noise (Supplementary Figure S4, S5).

Model and Parameter Recovery

We performed model and parameter recovery for a range of relevant parameter settings. Parameter recovery was performed for ten different synthetic internal models that spanned a range of hidden states (K, five different values) and a range of emission matrices (Supplementary Figure S3a-b). Variations in emission matrices explored different levels of stochasticity in the internal model: In odd states high probabilities were assigned to the originally deterministic observation (1, 0.95, 0.90, 0.85, 0.80), and a uniform probability was assigned to the rest of the potential observations; in the even states a uniform probability was assigned to each observations. Transitions between states in synthetic internal models were kept deterministic. Synthetic RTs were generated based on the synthetic internal models, and then internal models were inferred from the RTs by CT. We assessed the success of model recovery by contrasting the inferred number of states with the number of generative states (Supplementary Figure S3c). The number of inferred hidden states was assessed for synthetic response time data generated by the modified emission matrix (Supplementary Figure S3d). Learning of the parameters of the generative model through CT was assessed by contrasting the inferred parameter values with the ground truth parameters of the model underlying the synthetic response time data. We show the inferred emission probabilities for the range of tested generative emission probabilities (in order to avoid non-identifiability of parameters, we plot emission probabilities of those posterior samples that featured eight hidden states; Sup-

plementary Figure S3e). In order to assess the efficiency of model learning on the quantities that are directly relevant for quantities measured in experiments, we calculated the generative and inferred log predictive probabilities for a range of models (Supplementary Figure S3f). Correlations between the generative and the inferred log predictive probability values are reasonably high ($r_{M_{1.00}} = 0.894$, $r_{M_{0.95}} = 0.767$, $r_{M_{0.90}} = 0.482$, $r_{M_{0.85}} = 0.533$, $r_{M_{0.80}} = 0.419$, and p-values are all < 0.001***). Finally, we also performed a model identifiability analysis. For this, we used CT to infer models on data synthesized from hypothetical internal models characterized by different levels of stochasticity. The inferred models were then evaluated on the alternative synthetic data sets. Inferred models were evaluated through the likelihood of the test synthetic reaction times (Supplementary Figure S3g).

Additional model validations were performed with the same model in [1]. Briefly, for three example iHMMs that were inferred from response time data (after 640, 1280 and 2400 trials). CT was applied to these response time distributions and assessed the inferred model on both the marginal distribution of response times and the subjective probabilities of the Ground Truth model and CT-inferred models. Finally, we computed the r^2 statistics for the Ground Truth and CT-inferred models. These experiments confirmed that the CT-inferred iHMM was as effective in accounting for variances in response times as the Ground Truth models were. Further, our analysis has demonstrated that the recovered model was as effective in predictive response times as the synthetic model and subjective probabilities were shown to be accurately recovered from response times when response time noise levels were close to those measured empirically.

Measures for the Analyses

Correlation

Correlations reported in the main text are all measured by Pearson's product-moment correlation coefficient, denoted as r.

Learning Strength

Learning strength characterizes the predictive power of CT in excess of the initial inductive bias. As such, it is designed to characterize the knowledge acquired by training. We calculate learning strength by subtracting the variance explained by Markov from the variance explained by Cognitive Tomography.

Within and Across-days Prediction

To capture the dissimilarity of two internal models, we test both internal models on separate days as well as on the test blocks on the same day as the training was performed. We call within-day prediction if the internal models were tested on the same day as they were trained (i.e. Task 1 IM tested on Task 1) and across-days prediction if the internal models tested on the other day as they were trained (i.e. Task 1 IM tested on Task 2, Figure 5b). Training of the models occurs on the training blocks, as defined above, both in case of within and across-days predictions.

Relative Performance

Relative performance measures are used to compare the performance of distinct inferred models. Since the strength of internal models are expressed in terms of explained variance (r^2), relative performance measured as a difference in explained variance. In Figure 4d, 4e we compare the advantage of the corresponding internal models (within-day prediction) over the non-corresponding (across-days prediction) internal models. Thus, relative performance on Day 8 is computed as $r^2_{Task\,1\,IM\,(D8)} - r^2_{Task\,2\,IM\,(D8)}$.[2] Relative performance on Day 9 is computed as $r^2_{Task\,2\,IM\,(D9)} - r^2_{Task\,1\,IM\,(D9)}$. On Figure 5d, we compare the relative performance of Task 1 and Task 2 internal models on segments governed by the rule matching the rule the internal models were trained on (four segments, with five blocks in each segment) of the alternating task presentation on Day 10 (see Figure 5b). Consequently, predictive performance on Task 1 blocks is calculated as $r^2_{Task\,1\,IM\,(D10\,Task\,1)} - r^2_{Task\,2\,IM\,(D10\,Task\,1)}$,[3] and on Task 2 blocks it is calculated as $r^2_{Task\,2\,IM\,(D10\,Task\,2)} - r^2_{Task\,1\,IM\,(D10\,Task\,2)}$.

Alternation Score

Alternation score is computed in order to express participants' ability to switch internal models according to the alternating stimulus governing rule. The higher the score, the better the ability of the individual to alternate between internal models. To compute the alternation score, first we centered the Task 1 and Task 2 internal models' predictive performances to zero for each participant separately, then we computed the corresponding models' advantage over

[2]Task notation in the lower index shows on which task was the internal model inferred, while the day labels in the parentheses note the day on which the internal model performance was evaluated.

[3]Task notation as before, labels in the parentheses indicate the block segments' identity, on which the internal model performance was evaluated.

the non-corresponding for each identical block segments (as in case of the relative performance computation). Finally, scores are averaged for each participant across block segments.

Normative Permutation

Since the task sequence on Day 9 is a permuted version of the Day 8 task sequence, we can express the Day 9 task sequence as a specific permutation of the Day 8 sequence: $Task\ 2 = \sigma^*(Task\ 1)$. Following the same logic and applying the same permutation on the states of the inferred internal model, we can express a 'normative version' of the Task 2 internal model by permuting the Task 1 internal model: $Task\ 2\ IM = \sigma^*(Task\ 1\ IM)$. In practice, we applied a permutation on the emission matrix of the Hidden Markov model. As opposed to the 'normative permutation' which is motivated by the actual experimental manipulation, we can also create alternative permutations. The deterministic component of the sequence is four-element long that has $4! = 24$ permutations. Since the sequence is recurring, the exact positions of the observations doesn't matter (the deterministic component sequence of red-blue-gray-yellow is equivalent with the deterministic component sequence of blue-gray-yellow-red), thus there is $24/4 = 6$ possible rotation invariant sequence, one of that is identified with the Task 1 rule, another as the Task 2 rule, thus there are 4 remaining permutations, that we name as alternative permutations.

References

[1] Török B, et al. (2022) Tracking the contribution of inductive bias to individualised internal models. *PLoS Computational Biology* 18(6):e1010182.

[2] Bishop CM (2006) Pattern recognition and machine learning. *Springer google schola* 2:5–43.

[3] Gael JV, Saatci Y, Teh YW, Ghahramani Z (2008) Beam Sampling for the Infinite Hidden Markov Model. *Proceedings of the 25th international conference on Machine learning* pp. 1088–1095.

[4] Carpenter R, Williams M (1995) Neural computation of log likelihood in control of saccadic eye movements. *Nature* 377:59–62.

[5] Kim TD, Kabir M, Gold JI (2017) Behavioral/Cognitive Coupled Decision Processes Update and Maintain Saccadic Priors in a Dynamic Environment. *Journal of Neuroscience* 37(13):3632–3645.

Supplementary Figures

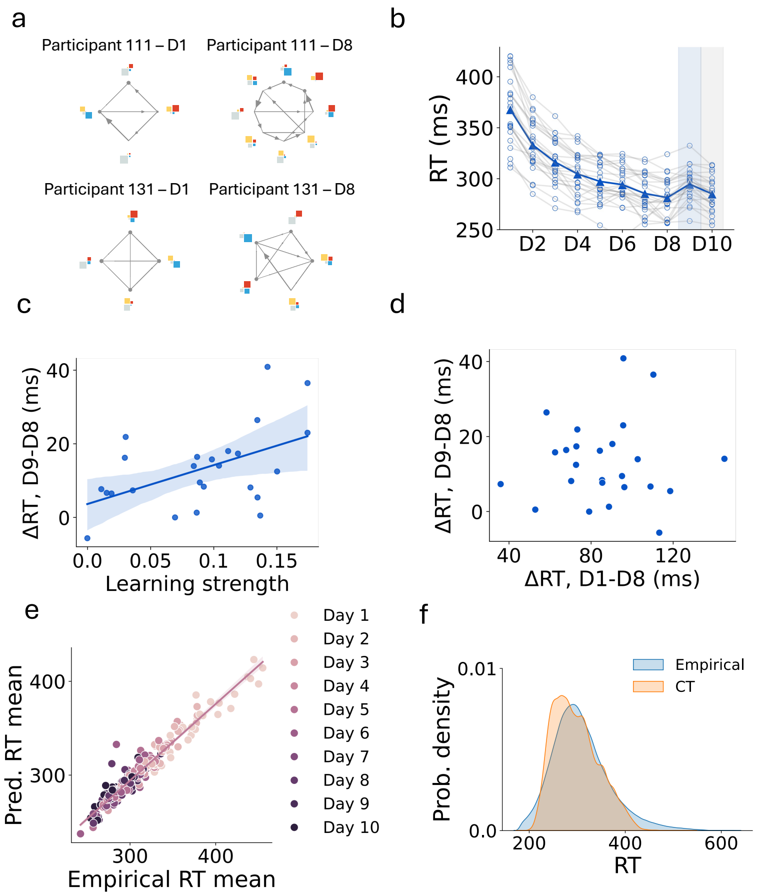

Figure S1: **a**, Example internal models for two participants on D1 and D8 of the Training Phase. Internal models are samples taken from the posterior inferred by CT. **b**, Evolution of response times (RT) during the experiment. Individuals (*thin lines*) and mean (*thick line*) are shown across the ten days of the experiment. **c**, Relationship between learning strength (measured in the D5-D8 sessions) and the increase in response time in the Transfer Phase (RT difference between D9 and D8). Dots indicate individual participants, line is a fitted linear, shaded area indicates 95% confidence interval. Response times on a given day are calculated as the mean over the 25 blocks of trials. **d**, Increase of response time in the Transfer Phase as a function of enhancement of mean response times across the first eight days of the experiment. Dots indicate mean RT differences for individual participants. **e**, Predicted response times show strong correlation with the empirical response times throughout the 10 days of the experiment ($r = 0.965$, p-value < 0.001***). Dots show individual participants' RT means on each day, line show the fitted linear, shaded area show 95% confidence interval. **f**, Comparison of empirical and CT-predicted response times for an example participant.

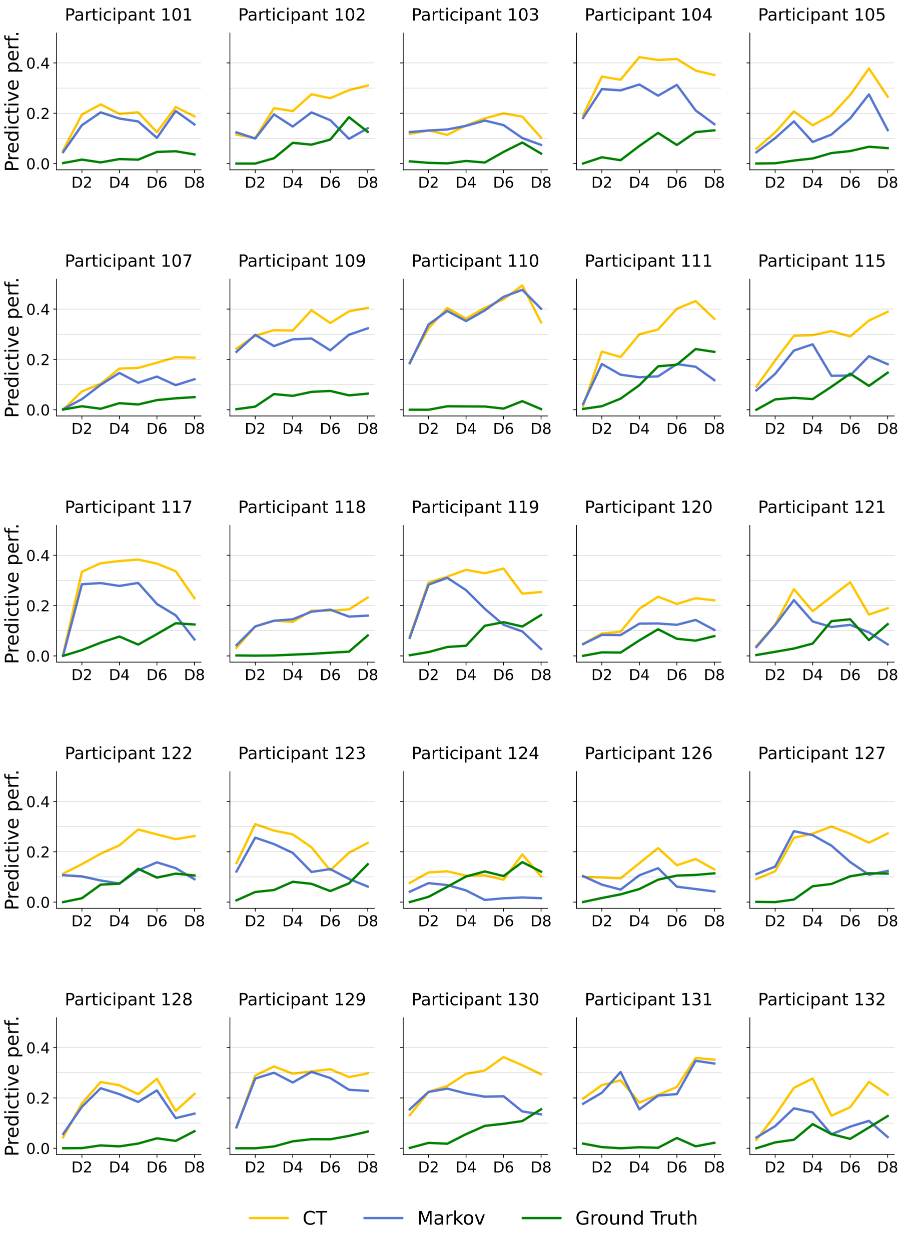

Figure S2: Learning curves for each participant during the Training Phase.

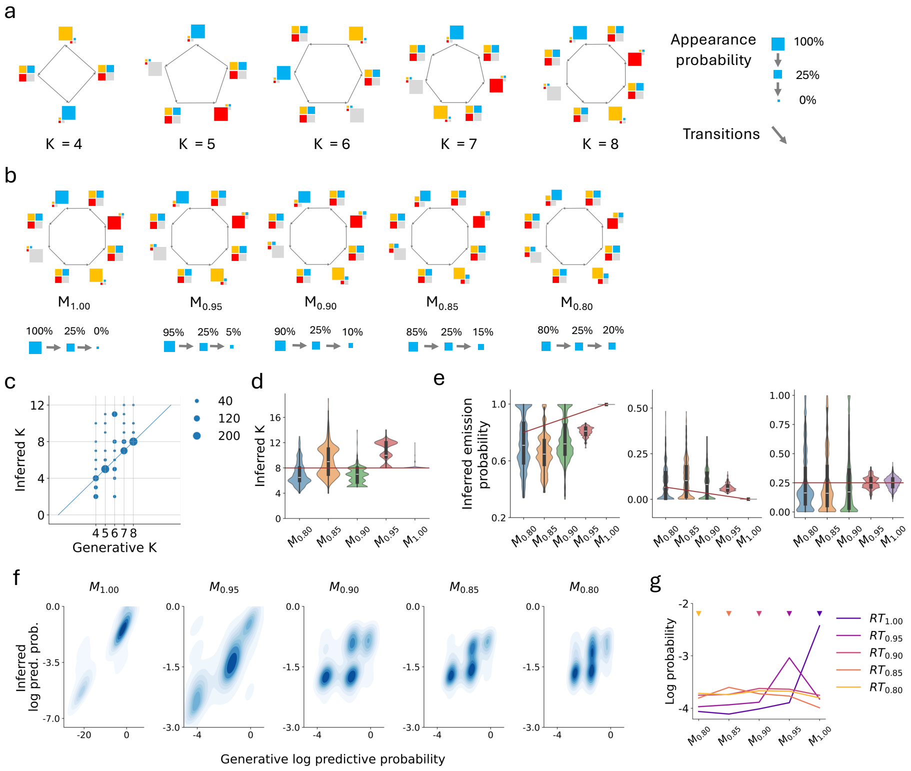

Figure S3: Model recovery and model identifiability. **a**, Schematics of the synthetic internal models parameterized by various number of hidden states (K), used for synthetic RT generation. Arrows denote transitions, square sizes show emission probabilities, i.e. probabilities of different observations associated to a particular state. **b**, Schematics of the synthetic internal models, parameterized by a range of different emission probabilities. Emission probabilities are not altered at the random states. Arrows and squares as in **a**. **c**, Recovery of the number of latent states for the five different generative models that feature different number of latents (as described in **a**, horizontal axis). Dot sizes denote the frequency of a given number of hidden states in the last 60 samples of four Markov chains that sample the posterior. In the case of perfect recovery, all data points would concentrate on the identity line for each generative K. **d**, Inferred K in the last 60 samples of five different generative models, distinguished by different emission probabilities (as explained in **b**). **e**, Inferred emission probabilities for five different generative models (only those samples are shown where the K = 8 was correctly inferred). The three panels show the three different emission types (from left to right): high, low and random. Expected values in case of perfect recovery shown by the brown line. **f**, Log predictive probability distribution for all trials in the synthetic and in the inferred models for five generative models, parameterized by different emission probabilities. **g**, Model identifiability. Model recovery was performed for five synthetic data sets generated by models with different emission matrices. Then, probability of synthetic reaction times, generated by the five synthetic models, were evaluated under each recovered model. Colored triangles indicate where each RT probability expected to peak, e.i. reach its maximum in the log probability space, in case of perfect identifiability.

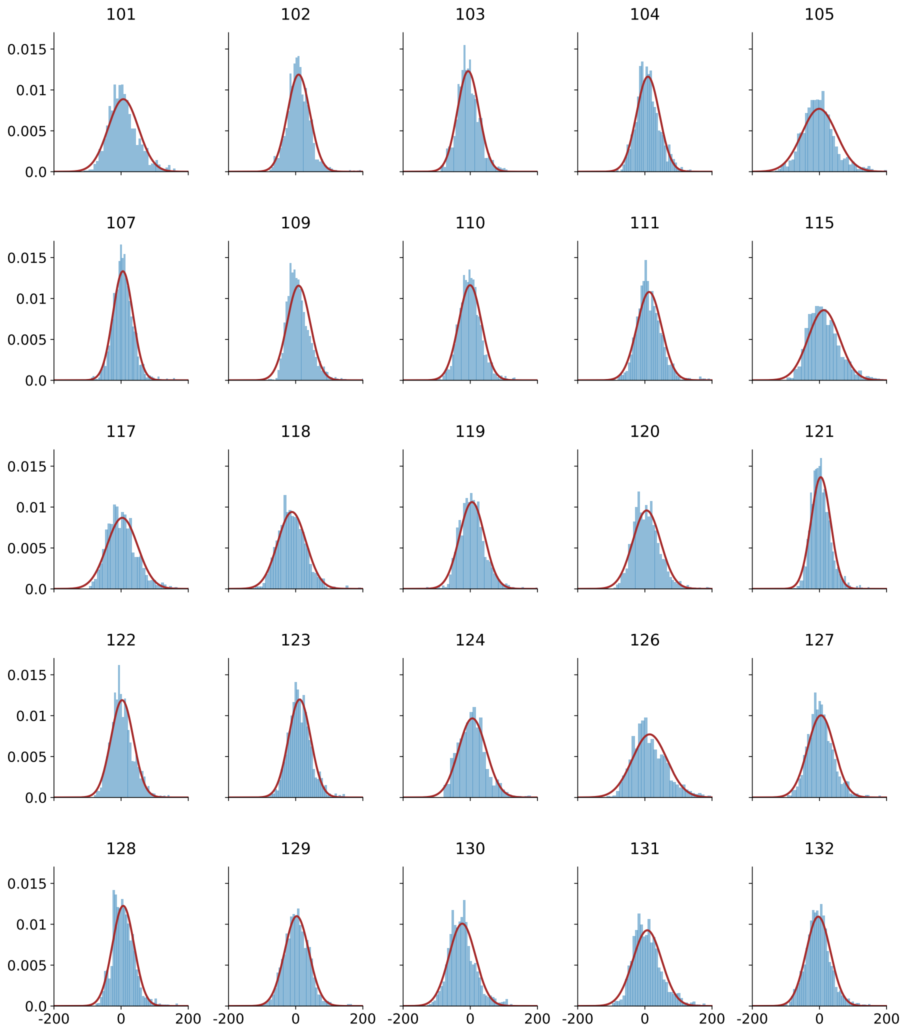

Figure S4: Residuals of response times in the CT model and Gaussian fits to the distribution on D8.

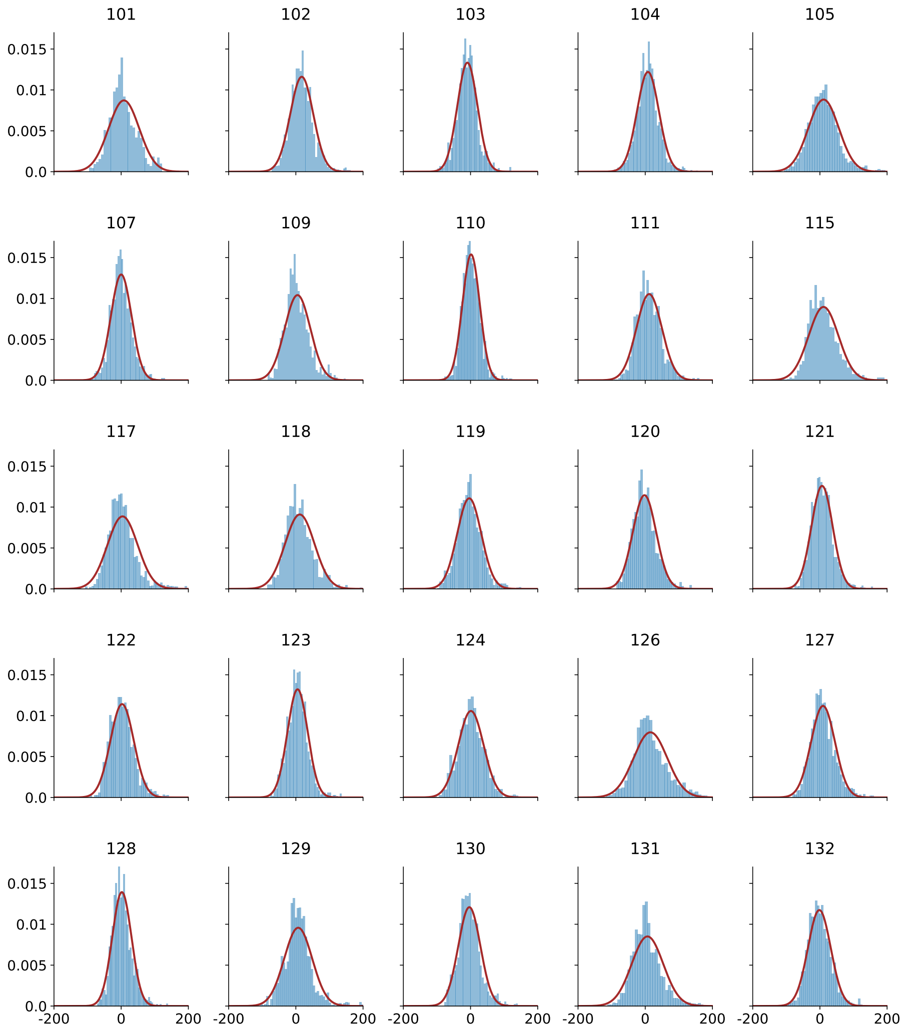

Figure S5: Residuals of response times in the CT model and Gaussian fits to the distribution on D9.
